# Living in the Flesh: Technologically Mediated Chiasmic Relationships (in Times of a Pandemic)

**DOI:** 10.1007/s10746-022-09625-7

**Published:** 2022-05-04

**Authors:** Bas de Boer, Peter-Paul Verbeek

**Affiliations:** Philosophy Group, University of Twente, Enschede, The Netherlands

**Keywords:** Merleau-Ponty, Flesh, Postphenomenology, Technological mediation, Chiasm, Coronavirus

## Abstract

During the Corona pandemic, it became clear that people are vulnerable to potentially harmful nonhuman agents, as well as that our own biological existence potentially poses a threat to others, and vice versa. This suggests a certain reciprocity in our relations with both humans and nonhumans. In his *The Visible and the Invisible*, Merleau-Ponty introduces the notion of the *flesh* to capture this reciprocity. Building on this idea, he proposes to understand our relationships with other humans, as well as those with nonhuman beings as having a *chiasmic* structure: to sense, or perceive another entity in a particular way simultaneously implies to be sensed or perceived in a particular way by this other entity. In this paper, we show how a postphenomenological perspective expands on Merleau-Ponty: first, it more radically interprets Merleau-Ponty’s notion of flesh by not only considering it to be a medium that is the condition of possibility for vision but as pointing to the constitution of an intercorporeal field in which entities—both human and nonhuman—mutually sense one another. Second, it augments Merleau-Ponty’s thought by drawing attention to how technologies mediate chiasmic relations. This is clarified through the example of the facemask, which (1) reveals the chiasmic structure of our relation with nonhuman entities, and (2) shows that technologies co-constitute interpersonal relationships by making humans present to one another in a particular way. We suggest that these aspects are not unique to the facemask, but point to a general *technologically mediated chiasmic structure* of human-world relations.

## Introduction

If there is one thing that the COVID-19 pandemic painfully reveals, it is that no individual escapes being a physical body that is vulnerable to a potentially harmful biological agent, as well as that one’s own biological existence might pose a threat to the vulnerable bodies of others. Technologies play a central role in the ways in which this vulnerability takes shape. While biological agents continuously affect our bodies, they often remain invisible; only through complex technological processes can such agents be identified and recognized as being particular ones such as COVID-19. Moreover, technologies are needed to communicate the effects of a biological agent on a society (e.g., graphs of death rates), and to protect human beings against the biological agent (e.g., facemasks and contact tracing apps).

In this article, we will investigate this technological mediation of bodily vulnerability. We will do so by connecting the postphenomenological approach to technology with Merleau-Ponty’s notion of the chiasm. One of the central ideas of postphenomenology is that we encounter the world and the objects existing in it only in relation to the technologies we use (e.g., Ihde, 1990; Verbeek, 2005). These technologies, then, mediate how we understand and experience these objects, how we interact with them, as well as the kind of human beings we take ourselves to be (e.g., Aagaard, 2020; Aydin & de Boer, 2020). For example, without the availability of technologies to observe coronaviruses and statistical techniques to model how they spread, the specific protective measures taken to mitigate its effects would not be possible. Moreover, the technologically mediated way in which the coronavirus enters society also shapes how human beings relate to other human beings and to nonhuman entities. For example, the facemask, a technology that explicitly serves to protect one’s own body and the bodies of others in the public sphere, reveals how people actively relate to both their own body as well as to the bodies of others as being vulnerable to a biological agent. At least potentially, a facemask wearer simultaneously makes visible her awareness of being an object that is impacted by a biological sphere and that can impact other biological beings; facemask users position themselves in terms of their own vulnerability and the vulnerability of other subjects.

In *The Visible and the Invisible*, Merleau-Ponty has argued that the twofold way in which one’s body can appear reveals that subjects and objects should not be separated but are always enveloped into one another. He argues that to be able to see, my own body must be part of the visible, in order to touch, it must be part of the touchable, such that when other beings become available to me, I become also available as visible or touchable to these other beings (Merleau-Ponty, 1968: 136). This reciprocity is possible, so Merleau-Ponty holds, because the beings that become visible in the acts of seeing, touching, etc. participate in the same medium: *the flesh* (*la chair*). Building on this idea, we propose to understand our relationships with other humans, as well as those with nonhuman beings (such as the coronavirus) to have a *chiasmic* structure: to sense, or perceive another entity (be it human or nonhuman) in a particular way simultaneously implies to be sensed or perceived in a particular way by this other entity.

It is our aim in this paper to show how Merleau-Ponty’s notion of “flesh” and “chiasm” are relevant when analyzing how facemasks shape relationships between humans, between nonhumans, and between humans and nonhumans. This analysis serves two theoretical purposes: on the one hand, we draw from postphenomenology to propose a more radical interpretation of Merleau’s Ponty’s idea of the chiasm. We develop the concept of *technologically mediated chiasmic relationship* to highlight that the notion of chiasm can be extended to how entities interact with one another beyond the human sphere and highlighting the mediating role that technologies play in constituting chiasmic relationships. On the other hand, we suggest that the reciprocal character of human-world relationships is insufficiently recognized within postphenomenology, and that Merleau-Ponty’s notions of “flesh” and “chiasmic relation” can help clarifying this reciprocity.

Our paper is structured as follows: First, we briefly situate our postphenomenological point of departure as a way of approaching the relations between humans and technologies (1st section). Second, we introduce Merleau-Ponty’s notion of the flesh, and the idea of the chiasm it gives rise to (2nd section). Third, we propose a more radical interpretation of Merleau’s Ponty’s idea of the chiasm by suggesting that it extends to how entities interact with one another beyond the human sphere and highlighting the mediating role that technologies play in constituting chiasmic relationships (3rd section). This we clarify through how technologies mediate chiasmic relationships by analyzing how wearing a facemask mediates relations between humans and nonhumans (4th section), and interpersonal relations (5th section). Then, we show how the notions of “flesh” and “chiasmic relation” can be integrated into a postphenomenological framework, and reveal that our relationship with technologies has a *technologically mediated chiasmic structure* (6th section). We conclude by suggesting the usefulness of the introduced concepts for analyzing human-technology relations beyond the issue discussed in this paper (7th section).

## Postphenomenology and the Notion of Technological Mediation

Let us start with a brief explanation of the distinct way in which postphenomenology attempts to analyze the relationships between humans and technologies.^1^ In postphenomenology, the notion of “technological mediation,” is used to capture that technologies are no neutral intermediaries allowing to better execute pre-defined goals and projects, but actively mediate how the world becomes present to us (e.g., Ihde, 1990; Verbeek, 2005). Accordingly, technologies are understood as shaping how individuals experience and understand themselves and the world in which they are situated. For example, the telescope instantiated a novel relation with heavenly bodies, leading to a rethinking of the place of human beings in the cosmos (Ihde, 2016). And more recently, brain imaging technologies make it possible to develop new ways of understanding human behavior (e.g., de Boer, 2020; Ihde, 2009), and ultrasound imaging changes norms about what is considered good parenthood (e.g., Verbeek, 2008). Also on a more general level, technologies texture the background against which we act, with digital technologies shaping educational practices (e.g., Aagaard, 2020), and algorithms mediating how the world can be understood and what kind of actions stand out as relevant (e.g., Wellner, 2018).

The concept of “technological mediation,” then, intends to capture how technologies mediate human-world relationships by helping to constitute specific forms of intentionality. Technologies, so it is proposed, do so by presenting or texturing the world in a specific manner, thereby simultaneously giving rise to specific forms of embodied experience and self-understanding.

In the next two sections (Sects. 2 and 3), we develop the idea that technologies mediate relationships between humans, between nonhumans, and between humans and nonhumans, and that these mediated relationships have a chiasmic structure. The goal of doing so is to highlight the reciprocal character of human-technology relations thus far largely neglected in postphenomenology, as well as to expand the postphenomenological work beyond its current focus on *human*-technology relations. In the next section, we start developing our proposal in detail by introducing Merleau-Ponty’s notion of *flesh*. This notion highlights the ontological interconnectedness of the human body and the world that structures any describable human-technology relation. We argue that the identified ontological interconnectedness points to the—thus far in postphenomenology largely neglected—*chiasmic* nature of human-technology relations. How such chiasmic relationships concretely manifest is clarified—later on in this paper—through the example of the facemask (Sects. 4 and 5).

## Merleau-Ponty’s Notion of Flesh and the Chiasmic Structure of Perception

In *The Visible and the Invisible*, Merleau-Ponty attempts to show how perception is possible by articulating the intimacy between seer and seen, touching and tangible, which is, according to Merleau-Ponty, “an intimacy as close as between the sea and the strand” (Merleau-Ponty, 1968: 130f.). In doing so, he intends to break through the idea that vision consists of a connection between an isolated and empty object that is completely identical with itself, and a seer who is similarly empty and opens herself to the visible to be filled by it in the act of seeing. However, taking as a point of departure the idea that seer and seen are enveloped into one another, he needs to give an account for why we neither completely blend into the visible, nor that the visible fully passes over into us, which would make vision “vanish at the moment of formation” (Merleau-Ponty, 1968: 131), due to the absence of any limit between seer and seen.

To sustain the idea that the touching and the tangible, and the seer and the seen are always interconnected with one another, but do not end up being completely identical, Merleau-Ponty introduces the notion of *flesh*. This notion articulates that in an act of vision, our look seems to be “in a relation of pre-established harmony with [the visible things], as though it knew them before knowing them, […] and yet the views taken are not desultory—I do not look at a chaos, but at things—so that finally one cannot say if it is the look or if it is the things that command” (Merleau-Ponty, 1968: 133). This relation of harmony makes it that perception is not an activity of the subject that allows for carving out specific visibles in an otherwise incomprehensibly chaotic field—this would be to introduce a form of transcendental subjectivity, and neither does the activity lie on the side of the object that independently carves out its own distinctive space of existence. Rather, there is a prior enmeshment of the seer and the seen: “he who sees cannot possess the visible unless he is possessed by it, unless he *is* of *it*” (Merleau-Ponty, 1968: 134f.). This “it” that both the seer and the seen “are of,” is what Merleau-Ponty terms the *flesh*.

In recent scholarship on Merleau-Ponty it has been repeatedly pointed out that the notion of flesh is highly ambiguous and has given rise to many misunderstandings (e.g., Alloa, 2017: 67; Carbone, 2015).^2^ One common misunderstanding has been to interpret the term “flesh” as being an equivalent to or generalization of the Husserlian notion of *Leib*. In *Ideas II*, Husserl famously distinguishes between *Leib* and *Körper*, and suggests that there is a unique double aspectivity to our body, namely that it is both a spatiotemporal object, as well as the very medium that makes it possible to experience other objects. On Husserl’s account, the body (*Leib*) appears as “the *medium of all perception,*” and a “zero point of orientation […] out of which the pure Ego intuits space and the whole world of the senses” (Husserl, 1989: 61). On this account, one is capable of entering into the world and grasping what is exterior on the basis of the subjectivity of one’s body that offers a medium of constitution. In one way or another, embodiment, here appears as something that is a pre-condition for entering in the world, but is itself not fully exterior. Merleau-Ponty’s late ontology of the flesh, however, intends to overcome this very duality between body (or consciousness) and world, such that the notion of “flesh” should not be equated with those of *Leib* or embodiment.

For Merleau-Ponty, it is not because we own our body that vision becomes possible, but rather that because we are part of the world that is made of certain stuff (flesh) that one can say that one has a body in the first place (see Alloa, 2017: 90). The flesh, then, is a condition for corporeality and the possibility of encountering other objects. Because our body belongs to the flesh, it has the capacity of seeing and being seen and encounter other visibles in virtue of this (Merleau-Ponty, 1968: 136). This can be clarified through the standard phenomenological description of an individual touching her own hand, an act in which both the individual’s capacity of touching and its capacity to be touched are exemplified. This capacity to be touched, or to be seen, reveals that a seer belongs to the same flesh that the seen is part of as well. It is through this shared material that vision becomes possible: “[T]he thickness of flesh between seer and the things is constitutive for the thing of its visibility as for the seer of his corporeity; it is not an obstacle between them, it is their means of communication” (Merleau-Ponty, 1968: 135). In the act of seeing, our body thus communicates with other sentient beings and in doing so distinguishes it from them as being sensible to other sentients. Yet, this is only possible because of their mutual belonging to the universal flesh of the world (Merleau-Ponty, 1968: 137).

Since our body belongs to the flesh as a sensible sentient, it is impossible to gaze over other entities, because it is the very belonging of the body to the flesh that makes vision possible: “My body as a visible thing is contained within the full spectacle [of vision]. But my seeing body subtends this visible body, and all the visible with it. There is reciprocal insertion and intertwining of one in the other” (Merleau-Ponty, 1968: 138). Through this reciprocity, the visible passes over into the seen, just as the seer is constituted as to be seen for others in the act of vision. In the act of seeing, the seen and the seer thus intertwine in the sense of becoming coupled in a particular way of becoming present to one another. This is what Merleau-Ponty calls the fundamental narcissism of vision: “[S]ince the seer is caught up in what he sees, it is still himself he sees” (Merleau-Ponty, 1968: 139). But more profoundly, seeing is a passive activity, it is “to be seen by the outside, to exist within it, to emigrate into it, to be seduced, captivated, alienated by the phantom, so that the seer and the visible reciprocate one another and we no longer know which sees and which is seen” (Merleau-Ponty, 1968: 139). Hence, vision presupposes an intimate connection with other visibles, as well as a sufficient distance that makes it possible to see and experience something as being something other than oneself.

The coupling of seer and seen in the act of vision does, therefore, not result in a solipsism in which every organism inhabits its own world in the absence of any interaction. Instead, the fact that other bodies also are sensible sentients and share the openness of the flesh—a flesh that passes through all beings—makes any solipsism impossible: “What is open to us, therefore, with the reversibility of the visible and the tangible, is—if not yet the incorporeal—at least an intercorporeal being, a presumptive domain of the visible and the tangible, which extends further than the things I touch and see at present” (Merleau-Ponty, 1968: 142f.). The notion of *flesh*, therefore, denotes a participation of all visibles in a shared medium in which they are constituted in a particular way in relation to others through how visibles are constituted for them.

Even though all entities participate in the flesh, their individual constitution might diverge. What the flesh generates is a certain openness for one another that is a condition of possibility for interaction. Before entering in a particular relation, “[t]here are two caverns, two opennesses, two stages where something will take place—and which both belong to the same world” (Merleau-Ponty, 1968: 263). However, *how* these entities are open to another is dependent on what a specific openness is responsive to, as well as how it is molded in its relation with another entity to which it has been opened up to. That is, the openness of a certain entity is what makes it possible to enter in a particular relationship with another one (and vice versa), but the different entities only attain a specific shape in virtue of being in a particular chiasmic relationship.

Also when entities are constituted in a particular way in a particular chiasmic relationship, they remain to be open to entering into novel relationships. If not, there would not be two entities in the first place, but a complete coincidence of them, which would turn them into *one* entity. Hence, the concept of “openness” functions to articulate that “[t]here is no coinciding of the seer with the visible” (Merleau-Ponty, 1968: 261). The openness makes it possible that each entity “borrows from the other, takes from or encroaches upon the other, intersects with the other” (Merleau-Ponty, 1968: 261). What this points to is that each entity, and importantly, each human being only takes a certain shape in its relation to other entities, and is constituted in virtue of how it is enveloped into other entities and the other way around.

No entity can be assigned certain characteristics or properties in isolation, but these are instead only constituted in the relationship with other beings. In understanding the relationships between entities in terms of a mutual constitution, Merleau-Ponty does away with any possible mechanistic causal explanation of the relationships with entities, as these kinds of explanations presuppose the existence of already constituted entities that engage in particular interactions in virtue of them having certain essential characteristics (see Carusi & Hoel, 2014: 210). Instead, the multitude of entities at any given moment make up a complex field in which they become sensible to one another, leading to their individual constitution. This is why it can be said that Merleau-Ponty holds that the relations we have with other beings have a chiasmic structure: in the act of vision, seers make themselves available to the others to be perceived in a particular manner and vice versa. The consequence of this perspective is that also experience is crucially dependent on the chiasmic relationship with other entities.

This view aligns well with Alloa’s interpretation of Merleau-Ponty’s late ontology: how other entities are perceived and attributed meaning to is dependent on the field that is constituted through the mutual relationships between the entities that constitute it, and has an irreducibly singular, embedded, embodied, and situated dimension (Alloa, 2017: 89f.). As McWeeny has put it: “The ontological concept of flesh allows us to affirm the relationality and complexity of lived experience, which does not present beings as either mind or body, active or passive, self or other, oppressed or privileged, but as both of these aspects at the same time” (McWeeny, 2014: 277). However, as we will show in the next sections, this chiasmic structure not only relevantly articulates how lived experience is always grounded in the particular chiasmic relationships that humans engage in but also shapes how nonhuman beings became visible to humans and humans to nonhuman beings: both interpersonal relationships and relationships with nonhuman beings have a chiasmic structure.

## Technologically Mediated Chiasmic Relations: Facemasks, Intercorporeity and the Coronavirus

In this section, we introduce the idea that both interpersonal relations and relations between human and nonhuman entities have a chiasmic structure. Furthermore, we argue that the specific character of the chiasmic relation is mediated by technologies that structure *how* nonhuman and human entities become present to one another. This can be understood as a generalization of the claim of the American postphenomenologist Don Ihde, who holds that, in scientific practice, every change in the observed object correlates with a change in the observing subject, and vice versa (e.g., Ihde, 1998: 155).

Our proposal extends Merleau-Ponty’s work on how intercorporeity is constituted from within the flesh in two ways: First, it more radically interprets Merleau-Ponty’s notion of flesh by not only considering it to be a medium that is the condition of possibility for vision but as pointing to the constitution of an intercorporeal field entities—both human and nonhuman—interact with one another. Second, it augments Merleau-Ponty’s thought by drawing attention to how technologies mediate chiasmic relations, which makes it possible to address the potentially different ways in which entities become present to one another in these relationships.

Merleau-Ponty’s analyses in the *Phenomenology of Perception* of how technologies can be integrated into one’s embodiment are an important source of inspiration for postphenomenology. For example, throughout the work of Don Ihde, Merleau-Ponty’s analysis of the blind man’s cane remains to be a central reference point (e.g., Ihde, 1990: 40, 1991: 29, 2009: 36). Merleau-Pony notes that “the blind man’s cane has ceased to be an object for him, it is no longer perceived for itself; rather, the cane’s furthest point is transformed into a sensitive zone” (Merleau-Ponty, 2014: 144). Through habituation and the integration of the cane into one’s embodiment, the world and the object are observed through the cane, which in turn gives rise to new ways of observation and plans of action. This example shows how humans are capable “of altering [their] existence through incorporating new instruments” (Merleau-Ponty, 2014: 145). The central point that Ihde derives from Merleau-Ponty is that such incorporations reveal that perception “is not limited by the outline of my body or the surface of my skin” (Ihde, 1990: 40), and that the use of technologies in perception transforms what we experience (e.g., Ihde, 1991: 29f.).

However, references to Merleau-Ponty’s later ontology of the flesh are scarce. At one of the few places in which this work is discussed, Ihde argues that the notions of *flesh* and *chiasm* denote that our being in the world is always such that although there is always a multiplicity of entities that we can attend to, most of them reside in the background of our experience. Although we can potentially engage with those entities and those entities with us, humans do not have full control over the types of entities that they can engage in: “I am immersed in the surrounding world, but this immersion is as flexible and dynamic as the panorama about me. This is the chiasm, the intertwining of the flesh of which Merleau-Ponty spoke in his last interpretations of perception” (Ihde, 1990: 46). However, what Ihde, somewhat surprisingly, does not focus on is that the individual’s flexible and dynamic immersion in the flesh is shaped by the presence of technologies, and how this presence shapes the field within which relations between humans and technologies take place (see du Toit, 2020). The main importance of Merleau-Ponty for Ihde’s work remains that technologies can be incorporated within embodiment and how they might alter embodied experiences. However, he pays almost no attention to how technologies constitute a particular field within which experiences, interactions, and observations become possible in the first place.

As we will suggest below, Merleau-Ponty’s late ontology can enrich our understanding of technologies, because it enables to articulate how technologies modify how different entities can be open to another, and how they help constituting particular chiasmic relationships. In his essay *Eye and Mind* Merleau-Ponty discusses how the painter’s use of a mirror makes it possible to turn oneself into a visible object. He notes that “like all other technical objects, such as signs and tools, the mirror arises upon the open circuit [that goes] from seeing body to visible body. Every technique is a “technique of the body”. A technique outlines and amplifies the metaphysical structure of the flesh” (Merleau-Ponty, 1964: 168). As Hoel and Carusi explain, such a view implies that technologies act upon “the body schema [that] has the capacity to be modified or transformed, for instance, by training and learning new skills, and even more so when symbolisms and tools (each with their own ‘nonhuman’ mode of operation) are introduced into the circuit—hence the insistence on the expansive dynamic of the flesh” (Hoel & Carusi, 2018: 56). Technologies shape the bodies of individuals—bodies that have an openness towards these technologies because they modify the character of the chiasmic relationships within the flesh. This hints at the possibility that the notion of flesh does not—as Merleau-Ponty sometimes seems to suggest—point to a rigid and more or less harmonious structure that is hardly subject to changes, but instead that it is continuously modified through the introduction of new technologies. It, therefore, seems appropriate to start understanding technologies as being part of the flesh, as well as understanding the chiasmic relationships between entities as technologically mediated. As we will clarify below, these technological mediations both apply in the context of interpersonal relations and in the context of relations between humans and nonhumans.

One might object that it is a stretch to maintain that (a) humans become visible (and actionable) to nonhuman entities in a particular way that is mediated by technologies, and (b) that an entity such as the Coronavirus is a candidate for being a body that is part of what Merleau-Ponty calls intercorporeity or intercorporeal being. If these objections are correct, it would not make sense to speak of a *chiasmic* structure of human-technology-world relations, because there is a *radical asymmetry* in how objects are constituted for the subject than the other way around.

We take it that objection (a) is best understood as stating that nonhuman entities lack, in contrast to human ones, the capacity of *intentionality* in the sense that they do not understand or experience *how* another entity becomes present to it, because they lack a meaningful context that is generative of experience and interpretation.

A first response to objection (a) is that not every nonhuman entity is the same. For example, it might not be too counterintuitive to say that certain mammals experience and have a certain sense of understanding of how other entities become present to them (Shew, 2017), whereas this seems less likely for, say, a walnut. We are not going to settle here which relations with nonhuman entities can be considered chiasmic and which cannot be, but rather would like to point out that it does not seem too much of a stretch to say that at least *some* relations with and between nonhumans can be considered to be chiasmic—and as we will suggest below, the human relation with the coronavirus amongst them.

A second more substantial response to objection (a) is that if one buys into the premises of postphenomenology—or technological mediation theory (e.g., de Boer, 2020; Verbeek, 2005, 2011), intentionality is not a property of either subject or object through which certain relations are constituted. Instead, postphenomenology is grounded in a *relational ontology* such that entities—including humans—only exist in a particular way through their relation with other ones: existence is always *mutual* existence (e.g., Rosenberger & Verbeek, 2015: 19–21). Therefore, every relation precedes and is constitutive of its relata. And crucially, since most (if not all) relations are constituted in interaction with technologies, what the relata are, how they exist, and how they become available to one another, is technologically mediated. As a result, technologies are co-constitutive of *how* particular chiasmic relations are constituted.

This brings us to objection (b): according to Merleau-Ponty, it is precisely because humans have a body that they are enveloped into the flesh. *Having* a body is therefore constitutive of intercorporeity. The core of objection (b) can be put as follows: nonhuman entities such as the Coronavirus cannot be said to have a body in a relevant sense, such that they cannot be understood as *sensible sentients*. Therefore, these entities are no candidates for being interpreted in terms of intercorporeal existence, making chiasmic relations with them impossible. We take it that this second objection is best replied to by means of a concrete example that shows that also relations between nonhumans, as well as the human relation with the Coronavirus is both chiasmic and technologically mediated.

## Technologically Mediated Chiasmic Relations with Nonhumans: the Facemask and the Coronavirus

As we saw, Merleau-Ponty characterizes the human being as a sensible sentient that has the double capacity of engaging both in the activity of *sensing* and in passively *being sensed*. In his discussion of intercorporeity, Merleau-Ponty already suggests that the reciprocal intertwinement of chiasmic relationships might not be limited to human bodies. He notes that also in the relations between nonhuman organisms, it can be said that “their landscapes interweave, [and] their actions and their passions fit together exactly” (Merleau Ponty, 1968: 142). Through the example that follows, we intend to clarify in more detail that the reciprocal intertwinement present in chiasmic relations is not peculiar to relations between humans, but extends to relationships between nonhumans, as well as to those between human and nonhumans. Furthermore, our discussion of how the facemask and other technologies are used to develop a specific relationship with the coronavirus serves to show that our chiasmic relation with it is *technologically mediated*. In the next section, we show how the same chiasmic structure also applies to interpersonal relations—albeit slightly different.

An example of a chiasmic relationship between nonhumans is that between an ant and the deadly fungus *Ophiocordyceps unilateralis*. This fungus is one of the most (in)famous manipulators of animal behavior. It infects the ant, which, once infected, is forced to climb up a near plant and clamp its jaws around the plant, waiting there until it dies. Then, mycelium grows out of the ant’s feet which stitches it to the plant’s surface, sprouts a stalk out of its head, from which infectious spores spoil down on the other ants passing below. Eventually, then, these other ants also end up being infected and manipulated into similar behavior, all contributing to the growth and survival of the fungus (e.g., Sheldrake, 2020: 107–109).

What this indicates is, to use Merleau-Ponty’s terminology, that an ant and fungus have a mutual openness to one another that makes it possible to enter in a certain relationship (one that, in this case, leads to the death of the ant). Put differently, initially, ants and fungus are capable of *sensing* one another. However, the *structure* of this chiasmic relationship is neither fixed nor symmetrical. It changes over time, and it is clear that the body of the fungus has a clear advantage over that of the ant. The example reveals two things that are highly relevant for understanding the relationship between humans and the Coronavirus, and which significantly expand Merleau-Ponty’s discussion of intercorporeity and the flesh: (1) also nonhuman bodies can enter into chiasmic relationships in virtue of them participating in the flesh, and (2) chiasmic relations should not be naively interpreted in terms of a harmonious relationship between two bodies, at least not when harmony is conceived of in terms of that the relationship must be *beneficial to* both bodies.

Important to note here is that the ant in this case proves to be extremely *vulnerable* to the fungus. However, and this marks an important difference between the example of the ant-fungus relationship and the human relationship with the Coronavirus, human beings are capable of rapidly altering the relationship with the Coronavirus through technological interventions.^3^ For example, the development of a successful vaccine would alter the relation, but changes can also manifest through other ways in which humans texture the environment technologically. Now, how can we interpret the relationship between human beings and the coronavirus as chiasmic?

Just as in the relationship between fungus and ant described above, humans and the coronavirus can sense one another, both as passively sentient and as actively sensible. For example, a human body is an environment that can be sensed by a virus as one that is beneficial for its survival, which in turn makes it that the human body can start sensing the virus by noticing its effects (e.g., sore throat, fever, feelings of breathlessness). In this case, the virus *actively* senses the human body, simultaneously constituting the human body as *actively* sensing its being affected by the virus. When noticing its being infected, the human body might activate its immune system, as a result of which the relationship with the virus takes a different shape again. Here, the virus is related to as an intruder that should be fought, in contrast with the relationship with other microorganisms that can be beneficial to the human body (consider for example the human microbiome). In this case already, it can be seen that how the human body becomes present to the virus, is constituted through how the virus becomes present to the human body and vice versa.

The mutuality of sensing becomes significantly clearer when not reducing the interaction between the human being and the virus to a physiological one. After all, when one recognizes or suspects being infected with the virus, one likely searches for opportunities for shaping the relationship with the virus in a particular way. Put differently, the structure of this chiasmic relationship is such that the human being actively starts seeing the virus as a risk to her health status. When *sensing* to be in a particular chiasmic relationship, a desire for change can arise (i.e., the desire for being in another chiasmic relationship). For example, one might search for medical care in order to treat the complaints resulting from a particular chiasmic relationship in which the human body senses its vulnerability to the entity being related to. In doing so, through its passively being sensed as an environment in which the virus can remain to survive, humans are simultaneously constituted as actively perceiving the virus as something that it is vulnerable to.

This brings us to the second point: the technologically mediated nature of chiasmic relationships. The current pandemic reveals that the technologies used to shape the relationship between humans and the coronavirus, ranging from “mundane” ones such as facemasks or arrows used in shops to designate a specific walking direction, to “advanced” ones such as those present in the IC departments of hospitals. And finally, the vaccine against the current virus can be considered a technology *par excellence* that shapes how humans relate to the coronavirus by mitigating their vulnerability to it.

As these examples indicate, one of the ways in which the chiasmic relationship between human beings and the virus is subject to change is through how human beings relate to technologies. Due to the recognition of being vulnerable to the coronavirus, the material environment in which humans live has significantly changed as a result of technological interventions. Clear examples of this are calls for social distancing or the wearing of facemasks in public areas. Both of these measures are technological interventions that were motivated by sensing the human vulnerability to the virus, in order to reshape the chiasmic relationship with the virus by making humans *less vulnerable* to it. That is, how humans and nonhumans mutually sense one another (a) *shapes* the technological infrastructure in which humans live, and (b) *is shaped* by the technologies humans surround themselves with.

Now, how can the shaping—or mediating—role of technologies in the chiasmic relationship with the Coronavirus best be understood? Let us briefly clarify this by focusing on the facemask. The logic behind wearing facemasks is grounded in the idea that our relationship with the Coronavirus becomes modified when wearing them: it helps prevent the virus from spreading, thereby contributing to the decrease of the number of individuals vulnerable to the virus. The use of facemasks themselves is in itself not novel; protective masks have always been used in specific circumstances. For example, dentists, surgeons, or construction workers in a polluted environment have been routinely wearing facemasks way before the current pandemic (e.g., Leone, 2021). Through the widespread use of facemasks, the chiasmic relationship between humans and the virus changes because both start relating to a common third (i.e., the facemask). This common third shapes how the human being and the coronavirus are constituted, thereby also shaping how they mutually sense one another. In this case, it makes it more difficult for the coronavirus to spread, due to a change in how it senses the human being, accompanied with a change in the human being in its capacity of being sentient. Vice versa, the human being has a different way of sensing the coronavirus, because now it starts becoming visible as an entity being vulnerable to the technological measures being undertaken. Put differently, the facemask introduces a clear *asymmetry* in how the chiasmic relationship is constituted, and one which is one of the relata is being constituted differently; in this case, it puts the human in a less vulnerable position.

This asymmetry is constituted here because with the facemask a previously non-existing form of *transitivity* is introduced. Through the introduction of a particular form of transitivity, the chiasmic relationship does not disappear, but its particular structure is modified due to how the facemask mediates how human beings and the coronavirus mutually *sense* one another and how they *are sensed* by one another. Put differently, the structure of intercorporeity remains present, albeit in a different shape because the facemask mediates the possibilities for sensing and action for both entities involved in the relationship: it literally is a condition of possibility for the structure that a chiasmic relationship might take. And, as a result of the particular material constitution of the coronavirus, the human body, and the facemask, the structure of the chiasmic relationship is mediated now in such a way that the vulnerable properties of the Coronavirus stand out, rather than those of the human body.

## Technologically Mediated Interpersonal Relations

Not only the relations between nonhumans and between humans and nonhumans can be characterized as chiasmic, but the same holds for interpersonal relations. For Merleau-Ponty, also other humans are sensible sentients who are always constituted in a particular way in their reciprocal relation with others. As a result, it does not make much sense to speak of “the human being” in isolation, but only as a being that comes into being through the process of sensing others and being sensed by them. As we show in this section, technologies help to shape how this process takes place, by mediating how humans perceive and are being perceived by each other. The current use of facemasks, and the discussions surrounding them—we are writing this article in times of the COVID-19 pandemic—is a good example to illustrate this claim. Just as in the case discussed in the previous section, facemasks can be considered as a ‘common third’ that introduces a previously non-existing form of technological transitivity, mediating interpersonal chiasmic relations.

In order to analyze this mediated chiasm, we can again connect to the postphenomenological approach to human-technology relations. From the perspective of this approach, facemasks help to shape how human beings are constituted for each other, by mediating the hermeneutic relations that exist between them: facemasks reorganize how human beings can understand and be understood by each other. Several dimensions of this mutual interpretive relation are reconfigured when facemasks are used.

First of all, facemasks mediate the possibilities for *facial expression*. Persons wearing a mask cannot show their mouth and nose, which has profound implications for the possibilities to express oneself and to enable other people to read one’s intentions and emotions. Facemasks make it virtually impossible to smile to greet each other, to express fear or anger, disgust or surprise. Moreover, they limit nonverbal interpersonal communication to eye contact, which can help to express interest, empathy, or concern but which can also feel uncomfortable (Mheidly et al., 2020).

Second, facemasks mediate the *intimacy* of the contact between people. Their function to filter the air that people exchange makes explicit that people are actually exchanging air all the time. This intimate dimension of interpersonal relations, which usually remains implicit, becomes visible in a paradoxical way: on the one hand, instead of breathing each other’s air, people now wear a “ventilation condom” to immunize themselves for potential infections by others, and to prevent that others might be infected by them. Our shared air mediates our chiasmic relations as breathing beings: the air we breathe is breathed by others, and the air others breathe is breathed by ourselves. Facemasks reveal this intimacy, which largely remains unnoticed, by problematizing and “condomizing” it. On the one hand, however, wearing a facemask also enables one to express the intimacy between human beings and can reveal a willingness to actively care for it.

A third dimension in this mediated interpersonal hermeneutics concerns our *vulnerability*. Facemasks reveal ourselves and the other as vulnerable beings: as beings who can both be infectious and infectable. By protecting ourselves against infection, and others against being infected by us, facemasks add a profoundly ethical dimension to every interpersonal encounter. Emmanuel Levinas considers the face of the other to be the ultimate expression of someone’s vulnerability, and therefore the ultimate call for responsibility that makes it possible to enter into discourse with the other (e.g., Levinas, 1969: 201). The specific way in which facemasks *cover* the face has, from this perspective, a significant ethical significance. Facemasks do not reduce but rather enlarge the vulnerability of the other since they let us see and experience how others depend on the responsible behavior on our part, and the other way round (e.g., Ji, 2020). Again, the facemask can express an explicit recognition of vulnerability and the desire to behave responsibly in light of this (or be negatively interpreted by people that are skeptical about the current Corona measures as revealing a blind obedience to questionable governmental techniques, thereby signifying a lack of responsibility).

The mediated chiasm present in interpersonal relations finds its basis in a *mediated flesh*. The intercorporeity in facemask-mediated chiasmic relations takes on a thoroughly mediated shape: the mutual masking adds a dimension of intermediality to intercorporeity. The masked body, with its covered facial expression and condomized ventilation forms a shared condition for the ways in which covid-vulnerable humans are constituted for each other. Facemasks, therefore, become an intrinsic part of the flesh. Rather than being neutral additions *to* it they are constitutive elements *of* it. This “techno-flesh” is not just an addition of technology to the flesh: facemasks re-flesh the world, in the sense that they reorganize the shared material field within which entities can mutually sense one another. Facemasks do not only help to shape how human beings are constituted in relation to each other, but they also become part of the condition for this relation to take shape in the first place. Techno-flesh is not to be seen as a medium that exists between human beings. Instead, it is a new, technologically mediated form of intercorporeity, that conditions interpersonal relations in new ways.

## The Flesh as Techno-Flesh

We have suggested above that the Merleau-Pontian notions of “flesh” and “chiasm” point to an ontological interconnectedness prior to any describable human-technology relation. Seemingly, this suggestion stands in sharp contrast with earlier suggestions of postphenomenologists that focusing on the transcendental is a form of backward-looking and contains a metaphysical baggage presupposing that technology is something that human beings should be protected from (e.g., Verbeek, 2005). From this perspective, a transcendental analysis is incapable of laying bare the multiple and often surprising ways in which humans interact with technologies (e.g., Rosenberger & Verbeek, 2015). How, then, can these concepts become part of postphenomenology without introducing a form of back-word looking?

To clarify this, it might be helpful to recall one of Luce Irigaray’s criticisms of Merleau-Ponty’s work. She argues that he, in the *Visible and the Invisible*, mistakenly presupposes that all chiasmic relations are symmetrical and reversible, which makes him insufficiently able to account for relevant differences between and across chiasmic relations (Irigaray, 1993: 178; see Butler, 2008). That is, she takes the notion of chiasm to presuppose the existence of a universal whole in which both beings participate and can only meet on the condition of this participation. As a result, she argues, humans are only capable of seeing themselves in such a universe that is ontologically closed to the particular conditions of possibility for being in a chiasmic relation that Merleau-Ponty postulates. The suggestion here is that Merleau-Ponty’s account presupposes a universal structure to the flesh that culminates into a “refusal of otherness and openness” (Chanter, 2000: 231). On this account, a move towards the transcendental as Merleau-Ponty makes it, is a move backwards that closes off the possibility that particular technologies can be a source of novelty, difference, or disruption.

However, in the way we have elaborated upon Merleau-Ponty’s late ontology, a less universalizing picture emerges because we do not presuppose that chiasmic relations are necessarily symmetrical or reversible. When conceiving of flesh as a “techno-flesh” that is reconstituted anew due to the introduction of technologies that make possible new forms of transitivity, the notion of “flesh” no longer prioritizes a more authentic form of being-together that is endangered by technological developments, nor does it point back to a form of harmonious intercorporeity from which chiasmic relations originate. Rather, technologies constitute the field within which entities mutually sense one another anew. Even though any relation presupposes the possibility of mutual recognizing one another as a sensible sentient, thereby necessitating a chiasmic structure, the *way in which* these chiasmic relations concretely manifest themselves is dependent on both the particular characteristics of the technology involved, the ways in which the other entity is qualitatively apprehended as a sensible sentient (see Botin, 2021).

**Fig. 1:**
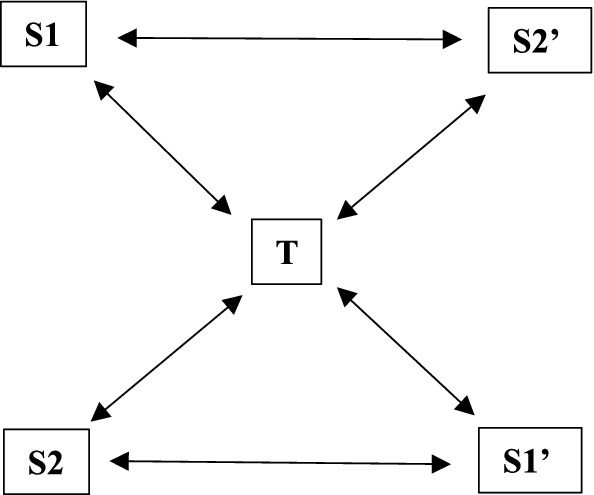
Technologically Mediated Interpersonal Chiasmic Relationship

Conceiving of chiasmic relations as such indicates that how an entity relates to another entity through a technology does not need to coincide with how this relation is constituted the other way around. In Fig. 1, this is schematically indicated by the fact that the lines between the two differently constituted subjects (S2 and S2’ or S1 and S1’) are interrupted (in this case through their relation with technology T), such that the subject S2’ that is related to by S1 does not coincide with the subject S2, and vice versa. The subjects in the different relationships appear as qualitatively different from one another, due to their becoming relative to both the particularities of the subject they are apprehended by and to their relations to the technology in question. As a result, the relationships S1 ←  → S2’ and S2 ←  → S1’ need not be, and most likely will not be, reversible or symmetrical, even though they are in a transitive relation with technology T.

Acknowledging the chiasmic nature of human-technology relations, therefore, does not imply a return to a backward-looking approach to technology that only focuses on the conditions of possibility of specific human-technology relations. Instead, it opens up a new domain of investigation: the ways in which chiasmic relations concretely manifest themselves. The concepts we introduced in this article are therefore to be seen as an addition to the vocabulary of postphenomenology, which makes it possible to capture new aspects of how human-technology relations are structured, rather than suggesting that technology use can be *reduced* to particular conditions of possibility. Instead, pointing to the chiasmic structure of human-technology relations makes possible a new way of *forward-looking*, namely one in which specific human-technology relations can start to be interpreted as being relative to other human-technology relations.

Simultaneously, when human-technology relations are understood as having a chiasmic structure, it becomes possible to recognize that the use of the same technology constitutes different forms of subjectivity and objectivity in different entities (human or nonhuman) that are potentially in tension. The same holds for situations in which entities relate to different technologies; these differences manifest relative to one another. We have attempted to clarify the new opportunities for analyzing human-technology relations from this perspective in the previous sections by pointing to how the facemask mediates the perceptual and hermeneutic structure of interpersonal relations, and by how it mediates the relation between human beings and the Corona-virus.

## Concluding Remarks

In this paper, we have shown how a postphenomenological approach to analyzing human-technology relations can be augmented through highlighting the ontological interconnectedness of human beings and the world using the Merleau-Pontian notions of “flesh” and “chiasmic relation”. Furthermore, we have shown how Merleau-Ponty’s notion of flesh can be radicalized by extending it to relations between nonhumans and between humans and nonhumans, as well as that technologies shape the flesh within which entities are enveloped. The flesh, so we have suggested can be understood as a “technoflesh,” where “techno-” refers to the flesh being malleable by technological developments that mediate chiasmic relations. The concrete forms of intercorporeity that are constituted within those relations are hence not fixed, but in constant development. This suggests that being enveloped in the flesh not only makes possible vision in a more or less harmonious manner—as Merleau-Ponty seems to conceive of it, but that our relationship with other entities changes due to how technologies reconstitute the flesh, leading to the emergence of newly structured chiasmic relations.

We furthermore argued that our proposal to understand human-technology relations as having a chiasmic structure points to the fact that in the relation with a certain technology, other entities necessarily are constituted in a particular way for humans, and vice versa. In other words, human-technology relations do never occur in isolation, but always relative to other human-technology relations. Through a postphenomenological analysis of how the facemask mediates the relation between human beings and the Coronavirus and interpersonal relations during the current pandemic, we showed how a particular form of intercorporeity comes into being that is mediated by the facemask. First, we showed how the facemask mediates the relationship between human beings and the Coronavirus by constituting a particular asymmetrical chiasmic relationship through which human beings are constituted as less vulnerable to the virus. The particular asymmetry of this relation is not fixed but subject to change, both by new technological developments such as vaccinations or by mutations of the virus. Second, we showed how also interpersonal chiasmic relations are constituted differently due to the introduction of the facemask, giving rise to new forms of intimacy, vulnerability, and expression.

Whereas the analysis in this paper has focused on the Coronavirus and the pandemic it gave rise to, another area in which the concepts introduced in this paper could show their usefulness is that of climate change. Contemporary thinkers such as Timothy Morton (2013) and Bruno Latour (2017) have argued—in significantly different ways—that dealing with climate change and mitigating its potentially devastating effects requires an explicit recognition of the mutual constitutiveness of humans and nonhumans, as well as the willingness to care for how also nonhumans constitute one another “behind the back of human beings”. Indeed, Latour goes as far as to say that the current pandemic and the way it put limits on social and economic activities, can help us think about “other means of entering the ecological mutation without a blindfold on” (Latour, 2020: 1). Blok (2019) has suggested that doing so requires to attend to the earth’s materiality independent of human relationality or intervention. The notions of “chiasm” and “techno-flesh” might help address how technologies shape the flesh of the earth, thereby shaping its very materiality and the conditions in which entities can mutually sense one another. Therefore, it seems, these concepts are potentially useful tools to address another global crisis that has yet to fully reveal itself.

## Footnotes

1 Given that the literature on postphenomenology is vast, we do not attempt to give an exhaustive overview of the variety of ways in which postphenomenologists analyze different human-technology relations, and neither give an account of the theoretical differences within postphenomenology. For an extensive introduction to postphenomenological research, see Rosenberger & Verbeek (2015).

2 An in-depth discussion of the ambiguity of the notion of flesh, the misunderstandings it gave rise to, and its relation to Merleau-Ponty’s project in *The Phenomenology of Perception* is beyond the scope of this paper. For a detailed analysis of such issues, see Alloa (2017: 66–81); Carbone (2015: 7–20); Landes (2013: 161–180).

3 We leave open here the question whether ants are also capable of technologically texturing their environment such that they become less vulnerable to the *Ophiocordyceps unilateralis.* For now, it suffices to say, that ants have thus far proven to be unsuccessful in protecting themselves against this particular threat.

## References

[CR1] Aagaard J (2020). Beyond the rhetoric of tech addiction: Why we should be discussing tech habits instead (and how). Phenomenology and the Cognitive Sciences.

[CR2] Alloa E (2017). Resistance of the sensible world: An introduction to Merleau-Ponty.

[CR3] Aydin C, de Boer B (2020). Brain imaging technologies as source for extrospection: Self-formation through critical self-identification. Phenomenology and the Cognitive Sciences.

[CR4] Blok V (2019). Nothing else matters: Towards an ontological concept of the materiality of the earth in the age of global warming. Research in Phenomenology.

[CR5] Botin, L. (2021). Thinking things and thinging thoughts. Our being with technology. In S. Lindberg, & H.R-. Roine (Eds.), *Technology, literary theory and philosophy*. New York: Routledge.

[CR6] Butler J, Olkowski D, Weiss G (2008). Sexual difference as a question of ethics: Alterities of the flesh in Irigaray and Merleau-Ponty. Feminist interpretations of Merleau-Ponty.

[CR7] Carbone, M. (2015). *The flesh of images: Merleau-Ponty between painting and cinema* (M. Nijhuis, trans.). New York: SUNY Press.

[CR8] Carusi A, Hoel AS, Coopmans C, Vertesi J, Lynch M, Woolgar S (2014). Towards a new ontology of scientific vision. Representation in scientific practice revisited.

[CR9] Chanter T, Evans F, Lawlor L (2000). Wild meaning: Luce Irigaray’s reading of Merleau-Ponty”. Chiasms: Merleau-Ponty’s notion of flesh.

[CR10] de Boer B (2020). How scientific instruments speak: Postphenomenology and technological mediations in neuroscientific practice.

[CR11] du Toit J (2020). Living in the age of the embodied screen. Indo-Pacific Journal of Phenomenology.

[CR12] Hoel AS, Carusi A (2018). Merleau-Ponty and the measuring body. Theory, Culture & Society.

[CR13] Husserl, E. (1989). *Ideas pertaining to a pure phenomenology and to a phenomenological philosophy: Second book: Studies in the phenomenology of constitution* (R. Rojcewicz, & A, Schuwer, trans). Dordrecht: Kluwer.

[CR14] Ihde D (1990). Technology and the lifeworld: From garden to earth.

[CR15] Ihde D (1991). Instrumental realism: The interface between philosophy of science and philosophy of technology.

[CR16] Ihde D (1998). Expanding hermeneutics: Visualism in science.

[CR17] Ihde D (2009). Postphenomenology and technoscience: The Peking university Lectures.

[CR18] Ihde D (2016). Husserl’s Missing Technologies.

[CR19] Irigaray, L. (1993). *An ethics of sexual difference* (C. Burke & G.C. Gill, trans.). New York: Cornell University Press.

[CR20] Ji P (2020). Masking morality in the making: How China’s anti-epidemic promotional video’s present facemasks as a techno-moral mediator. Social Semiotics.

[CR21] Landes DA (2013). Merleau-Ponty and the paradoxes of expression.

[CR22] Latour, B. (2017). *Facing gaia: Eight lectures on the new climate regime* (C. Porter, trans.). London: Polity Press.

[CR23] Latour, B. (2020). *What protective measures can you think of so we don’t go back to the pre-crisis production model?* (S. Muecke, trans.). Retrieved April 12, 2021, from http://www.bruno-latour.fr/sites/default/files/P-202-AOC-ENGLISH.pdf

[CR24] Leone M (2021). The semiotics of the anti-COVID-19 mask. Social Semiotics.

[CR25] Levinas, E. (1969). *Totality and infinity: An essay on exteriority* (A. Lingis, trans.). Pittsburgh: Duquesne University Press.

[CR26] McWeeny J (2014). Topographies of flesh: Women, nonhuman animals, and the embodiment of connection and difference. Hypatia.

[CR27] Merleau-Ponty, M. (1964). Eye and mind (C. Dallery, trans). In Wild, J. (Ed.), *The primacy of perception and other essays on phenomenological psychology, the philosophy of art, history and politics* (pp. 159–190). New York: Northwestern University Press.

[CR28] Merleau-Ponty, M. (1968). *The visible and the invisible* (A. Lingis, trans.). Evanston: Northwestern University Press.

[CR29] Merleau-Ponty, M. (2014). *The phenomenology of perception* (D.A. Landes, trans.). London: Routledge.

[CR30] Mheidly N, Fares MY, Zalzale H, Fares J (2020). Effect of face masks on interpersonal communication during the COVID-19 pandemic. Frontiers in Public Health.

[CR31] Morton T (2013). Hyperobjects: Philosophy and ecology after the end of the world.

[CR32] Rosenberger, R., & Verbeek, P.P-. (2015). A field guide to postphenomenology. In R. Rosenberger, & P.P-. Verbeek (Eds.), *Postphenomenological investigations: Essays on human-technology relations* (pp. 9–41). Lanham: Lexington.

[CR33] Sheldrake M (2020). Entangled life: How fungi make our worlds, change our minds, shape our futures.

[CR34] Shew A (2017). Animal constructions and technological knowledge.

[CR35] Verbeek, P. P-. (2005). *What things do: Philosophical reflections on technology, agency, and design* (R.P. Crease, trans.). Pennsylvania: The Pennsylvania University Press.

[CR36] Verbeek, P. P-. (2008). Obstetric ultrasound and the technological mediation of morality: A postphenomenological analysis. *Human Studies,**31*, 11–26.

[CR38] Verbeek, P. P-. (2011). *Moralizing technology: Understanding and designing the morality of things*. Chicago: The University of Chicago Press.

[CR37] Wellner, G. (2018). From cellphones to machine learning. A shift in the role of the user in algorithmic writing. In A. Romele, & E. Terrone (Eds.), *Towards a philosophy of digital media* (pp. 205–224). Cham: Palgrave Macmillan.

